# Post-diagnosis physical activity in relation to mortality among prostate cancer survivors: a systematic review and meta-analysis

**DOI:** 10.1007/s10552-026-02197-2

**Published:** 2026-06-25

**Authors:** Pauline Benker, Alina Hamann, Michael F. Leitzmann, Michael J. Stein

**Affiliations:** 1 Department of Epidemiology and Preventive Medicine, University of Regensburg, Franz-Josef-Strauß Allee 11, 93053 Regensburg, Germany; 2 Institute of Epidemiology, Helmholtz Zentrum München, German Research Center for Environmental Health (GmbH), Ingolstädter Landstraße 1, 85764 Neuherberg, Germany

**Keywords:** Post-diagnosis physical activity, Prostate cancer survivors, Mortality, Meta-analysis

## Abstract

**Background:**

Physical activity after cancer diagnosis may reduce mortality. However, systematic evidence for this association for men living with prostate cancer remains limited.

**Methods:**

We searched PubMed, Embase, and CINAHL data bases from start to June 2025. Random-effects meta-analyses estimated summary hazard ratios (HRs) and 95% confidence intervals (CI) for all-cause mortality and prostate-cancer-specific mortality to assess the association between post-diagnosis physical activity, measured in metabolic equivalents of task-hours per week (MET-hr/week), and mortality among prostate cancer survivors.

**Results:**

We included ten studies with more than 50,144 participants (one study did not report the number of prostate cancer cases included in the meta-analysis) and 28,044 deaths. Higher levels of post-diagnosis physical activity (≥ 7.5 MET-hr/week) were associated with lower all-cause mortality (HR = 0.69; 95% CI: 0.62–0.77), compared to lower levels (< 7.5 MET-hr/week). Meta-analysis of five studies showed an inverse association between post-diagnosis physical activity and prostate cancer-specific mortality (HR = 0.77; 95% CI: 0.66–0.89). Meta-analysis of four studies showed that moderate-to-vigorous physical activity was inversely associated with mortality (HR = 0.62; 95% CI: 0.51–0.74).

**Conclusion:**

Higher post-diagnosis physical activity, including activity at higher intensities, was associated with lower all-cause and prostate cancer-specific mortality among men living with prostate cancer. Our findings suggest that physical activity may complement cancer care for prostate cancer patients.

**Supplementary Information:**

The online version contains supplementary material available at 10.1007/s10552-026-02197-2.

## Introduction

Prostate cancer is one of the most common cancers among men; globally, approximately 1.5 million new cases occur each year. It is the fifth leading cause of cancer-related death in men with 375,000 prostate cancer deaths annually [1]. By 2040, The Lancet Commission on Prostate Cancer expects the number of new diagnoses to rise to 2.9 million and the deaths to 700,000 [2]. This underscores the importance of improving prostate cancer survivorship and supportive care.

Besides established screening methods and oncological treatment strategies, increasing attention has been given to modifiable lifestyle factors, such as physical activity, which potentially improve prognosis after diagnosis [3]. In general, physical activity improves cardiovascular, metabolic, and mental health, and in older adults helps maintain function and reduce frailty risk [4]. For many cancer patients, physical activity represents a promising supportive intervention, because it can positively influence treatment tolerance, symptom burden, and potentially overall survival. For example, Nader et al. (2024) showed that physical activity improves cancer-related fatigue and quality of life in prostate cancer patients and may have a positive influence on survival [5]. Based on such evidence, the World Health Organization (WHO) recommends that cancer survivors engage in at least 150 min of moderate or 75 min of vigorous physical activity per week [6].

Beyond these insights, a previous meta-analysis reported lower all-cause and prostate cancer–specific mortality among physically active prostate cancer survivors [7]. However, that analysis was only based on the four studies and did not specifically examine the potential survival benefits of higher-intensity physical activity following diagnosis.

In this systematic review and meta-analysis, we assessed the current evidence regarding the relation between post-diagnosis physical activity, including moderate-to-vigorous-intensity physical activity (MVPA), and mortality among men living with prostate cancer.

## Methods

### Literature search strategy

PubMed, Embase and CINAHL were searched from database inception until 23rd of June 2025 using the following terms: (“physical activit*” OR exercise OR “motor activity”) AND (cancer OR neoplasm* OR carcinoma OR adenocarcinoma OR sarcoma OR tumor* OR malignanc* OR “abnormal cells”) AND (survivors OR survivor OR survivorship OR patients OR patient) AND (mortalit* OR fatal* OR “death rate” OR survival OR recurrence OR progression OR outcome* OR prognos*). At this point, no cancer type was prespecified in order to ensure a comprehensive evidence base. We included synonyms for post-diagnosis physical activity, cancer patients and mortality. There were no restrictions by geographical region or date but we restricted the search to humans only. The reference lists of all included studies were screened manually for relevant studies. The e-alert notifications in PubMed captured additional articles until February 2026.

During study selection, analyses were restricted to prostate cancer to limit clinical heterogeneity and enhance interpretability of pooled estimates.

### Eligibility screening

Two independent reviewers (PB, AH) screened titles and abstracts of the search results. In case a study fulfilled the criteria of exposure (cancer patient or survivor), intervention (physical activity) and outcome (mortality), it was considered for full-text screening. When uncertainty of relevance remained, full-text screening was performed. Studies were excluded if they met any of the following criteria: full text or comparator group was missing, trials evaluated only short-term exercise interventions or reported deaths occurring during the intervention period, mortality outcomes were not reported as hazard or odds ratios, or the study design was neither observational nor a randomized controlled trial. Disagreements between reviewers were resolved by discussion and involvement of a third reviewer (MJS).

### Data extraction

We gathered information from the eligible studies on first author, year of publication, region, name of the study, cancer types, exposure, assessment of physical activity, number of participants, number of deaths, outcome, study type, and risk measure. Where possible, we used risk estimates for MVPA. In the absence of MVPA, total physical activity, recreational physical activity, or moderate physical activity was obtained. To harmonise different physical activity levels, we created two categories for our analysis. A high physical activity group which meets or exceeds the WHO recommendations of 7.5 metabolic equivalent hours per week (MET-hr/week) [6] and a low physical activity group which includes every level of physical activity below that threshold, including no activity. Weekly activity duration was converted into MET-h/week, applying a factor of 3 for moderate activity and a factor of 6 for vigorous activity [8].

### Study quality assessment

A single reviewer (PB) used the Newcastle–Ottawa Scale to assess the quality of each included study [9]. It ranges from zero (indicating low quality) to nine (indicating high quality) and combines the categories selection, comparability and outcome. Selection is worth a maximum of four points based on representativeness of the exposed cohort, selection of the non-exposed cohort, ascertainment of exposure and demonstration that the outcome of interest was not present at start of study. Comparability accounts for a maximum of two points, rating if the study is adjusted for age and other confounders. Lastly the category of outcome, worth three points, included assessment of outcome, length of follow-up time and adequacy of follow up of cohorts.

### Statistical analysis

To account for heterogeneity between included studies, estimates were combined only if they pertained to the same cancer type (prostate cancer) and outcome (all-cause mortality or prostate cancer-specific mortality. We performed random-effects meta-analyses using restricted maximum likelihood (REML) estimation. Hazard ratios (HRs) were log-transformed and standard errors were derived from reported 95% confidence intervals (CI) on the log scale.

Between-study heterogeneity was quantified using the *Q*-statistic and the *I*^2^-index, which describes the proportion of total variation across studies not attributable to chance. Following conventional thresholds, *I*^2^ values of 25%, 50%, and 75% were interpreted as low, moderate, and high heterogeneity, respectively [10]. Heterogeneity was further expressed by the estimated between-study variance *τ*^2^.

Meta-regression was performed to evaluate whether summary estimates differed according to physical activity type, study year and physical activity cutoff definitions. Additional meta-analysis included only studies with information on MVPA. Lastly, publication bias was assessed using funnel plots and formal tests (Egger’s regression and Begg’s rank correlation tests) when at least ten studies were available.

All analyses were conducted in R (version 4.5) using the metafor package. Two-sided *p* values < 0.05 were considered statistically significant.

The protocol was registered in PROSPERO under the registration number CRD420261326182.

## Results

**Fig. 1:**
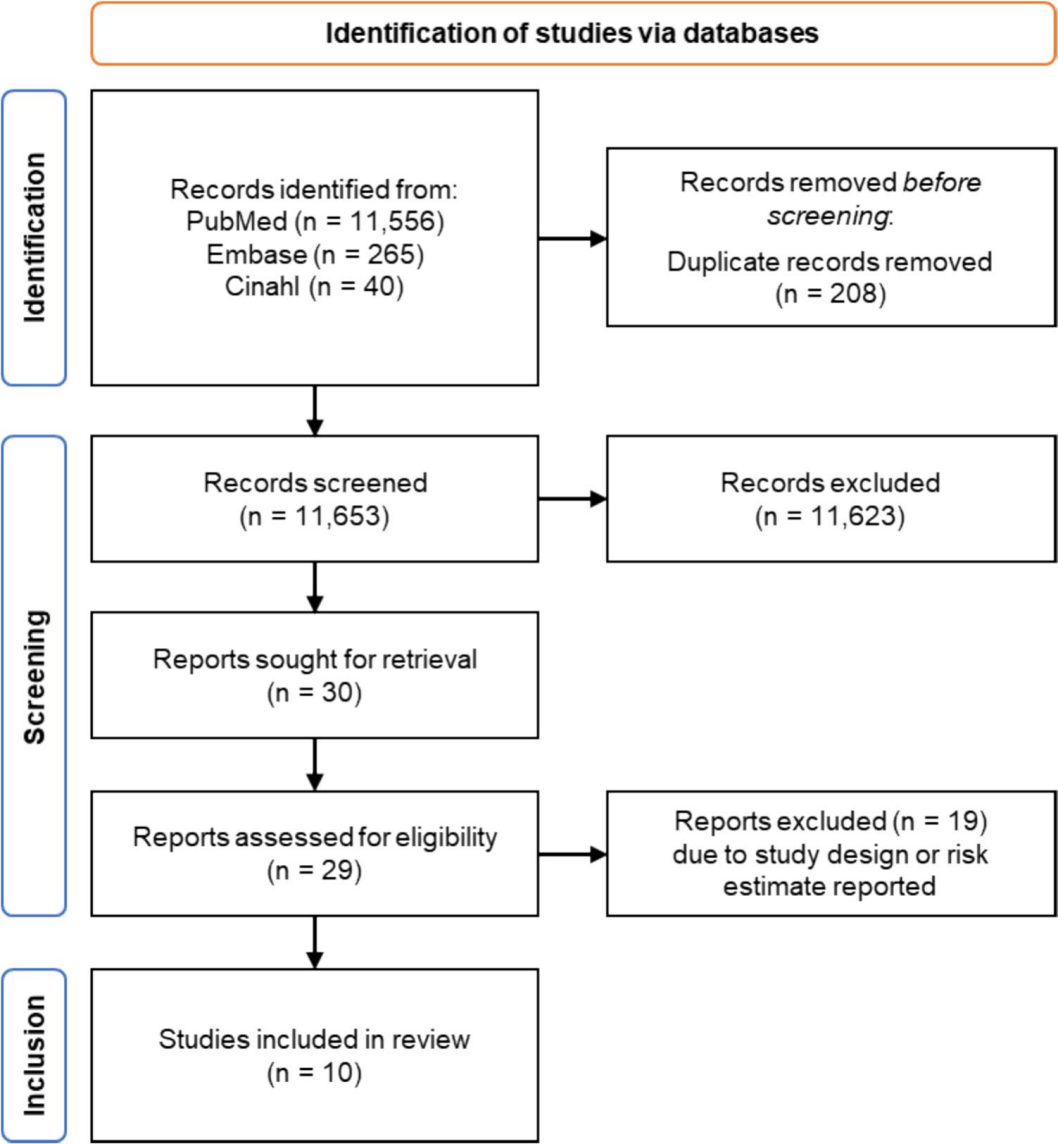
Flowchart of the screening and inclusion process

We identified 11,861 studies from the database search including all cancer entities. After duplicate removal and abstract screening, 622 studies remained. Of these, 29 studies were eligible and were identified as studies of prostate cancer populations. After full-text screening, ten studies were included in the analysis *(*Fig. 1*).*

### Characteristics of the included studies

The study characteristics of the ten included studies are listed in Table 1*.* In total, the study population comprised more than 50,144 participants (the sample size was unavailable from one study included in the analysis [11]). Across all studies, 28,044 deaths were reported during follow-up.

**Table 1:** Study characteristics of included studies

Author, Year, Country	Title of study	Number participants	Number deaths	Mean age at diagnosis in years	Median follow-up time in years	PA type	NOS Quality Score	References
Rees Punia (2025), US	Leisure-time physical activity after diagnosis and survival by cancer type: a pooled analysis	24,005	13,765	67,0	10,9	MVPA	9/9	[13]
Lavery (2024), US	Pan-Cancer Analysis of Post-Diagnosis Exercise and Mortality	3,980	1,590	68,0	16,0	MVPA	9/9	[15]
Tarasenko (2018), US	Muscle-strengthening and aerobic activities and mortality among 3 + year cancer survivors in the U.S	1,604	563	Categorized**	16,8	MVPA	9/9	[14]
Kenfield (2011), US	Physical activity and survival after prostate cancer diagnosis in the health professionals follow-up study	2,705	548	69,2	7,8 (dead)	Total PA	8/9	[18]
					9,7 (survivors)			
Friedenreich (2016), Canada	Physical activity and survival after prostate cancer	830	458	68,0	15,5	Recreational PA	9/9	[12]
Wang (2017), US	Recreational physical activity in relation to prostate cancer–specific mortality among men with nonmetastatic prostate cancer	5,319	1,685	71,0	6,8 (dead)	Recreational PA	8/9	[17]
					9,5 (survivors)			
Dickermann (2019), US	Guideline-Based physical activity and survival among US men with nonmetastatic prostate cancer	2,299	250	68,9	10,0	Moderate PA	9/9	[20]
Bonn (2015), Sweden	Physical activity and survival among men diagnosed with prostate cancer	4,623	561	63,1	4,7	Recreational PA	9/9	[16]
Laurberg (2024), Sweden	Diabetes-related risk factors and survival among individuals with type 2 diabetes and breast, lung, colorectal, or prostate cancer	360,260*	6,342	68,0	5,8	MVPA	8/9	[11]
Lee (2025), US	Postdiagnosis physical activity and dietary inflammatory and insulinemic potential with overall survival in men with nonmetastatic prostate cancer	4779	2282	69,0	15,0	Total PA	9/9	[19]

*360,260 includes all study participants diagnosed with prostate cancer. No detailed information is available on the number of the “cancer before diabetes diagnosis” group. In the analyses, the hazard ratio of the “cancer before” group was used

**in four age groups: 18–45 years (14,4% of all participants), 45–64 (35,7%),65–79 (34,5%), 80 + (15,5%)

*MVPA* moderate-to-vigorous physical activity, *NOS* Newcastle–Ottawa Scale, *PA* physical activity, *US* United States of America

All studies used prospective cohort designs, either multicenter or registry-based studies. Sample sizes ranged from 830 [12] to 24,005 [13] individuals.

Four studies reported post-diagnosis physical activity as MVPA [11, 13–15], three recreational [12, 16, 17] and two total physical activity [18, 19]. In most studies, physical activity was operationalized in MET-hr/week or categorized according to guideline thresholds. A few studies used categorized frequency or duration measures, such as times per week [14, 20] or sessions per week [11, 15].

Physical activity data were primarily obtained through self-administered questionnaires, in most cases with validated instruments, such as the Health Professionals Follow-up Study (HPFS) activity questionnaire, the HUNT 2 survey, or American College of Sports Medicine/American Cancer Society (ACSM/ACS) guideline-based tools.

Several studies performed detailed subgroup or sensitivity analyses. Friedenreich et al. [12] compared pre- and post-diagnostic activity levels. Bonn et al. [16] examined different activity domains and performed a 18-month lag analysis. Kenfield et al. [18] analysed both overall and prostate cancer-specific mortality with a 4–6 year lag time. Dickermann et al. [20] emulated a target trial to estimate hypothetical physical activity interventions. Lavery et al. [15] and Rees-Punia et al. [13] evaluated dose–response associations across four post-diagnostic activity categories (0, 0– < 7.5, 7.5 < 15, ≥ 15 MET-hr/week). Rees-Punia et al. further subdivided the highest category into three groups (15– < 22.5, 22.5– < 30, ≥ 30 MET-hr/week), allowing a more detailed examination of the upper activity spectrum. Lee et al. [19] considered both physical activity and the inflammatory and insulinemic potential of diet (assessed using rEDIP and rEDIH scores) and Laurberg et al. [11] conducted sub-analyses by cancer type within a nationwide diabetic cohort.

Of note, we identified one additional study that assessed post-diagnosis physical activity benefits stratified by cancer treatment [21]. The authors found effect modification by therapy: Those who received surgery or hormone therapy, but not those who received radiation therapy, had lower all-cause mortality if they engaged in higher versus lower post-diagnosis physical activity. Due to the absence of treatment-independent risk estimates, this study was not included in the meta-analysis.

Overall, the included studies consistently examined associations between post-diagnosis physical activity and all-cause or cancer-specific mortality. All studies were of high quality, scoring 8 to 9 points on the Newcastle–Ottawa Scale. The main reason for point deduction was the use of non-validated self-assessed questionnaires for the documentation of physical activity habits. Individual scores of each study are provided in *Supplementary Table A1*.

### Post-diagnosis physical activity and all-cause mortality

**Fig. 2:**
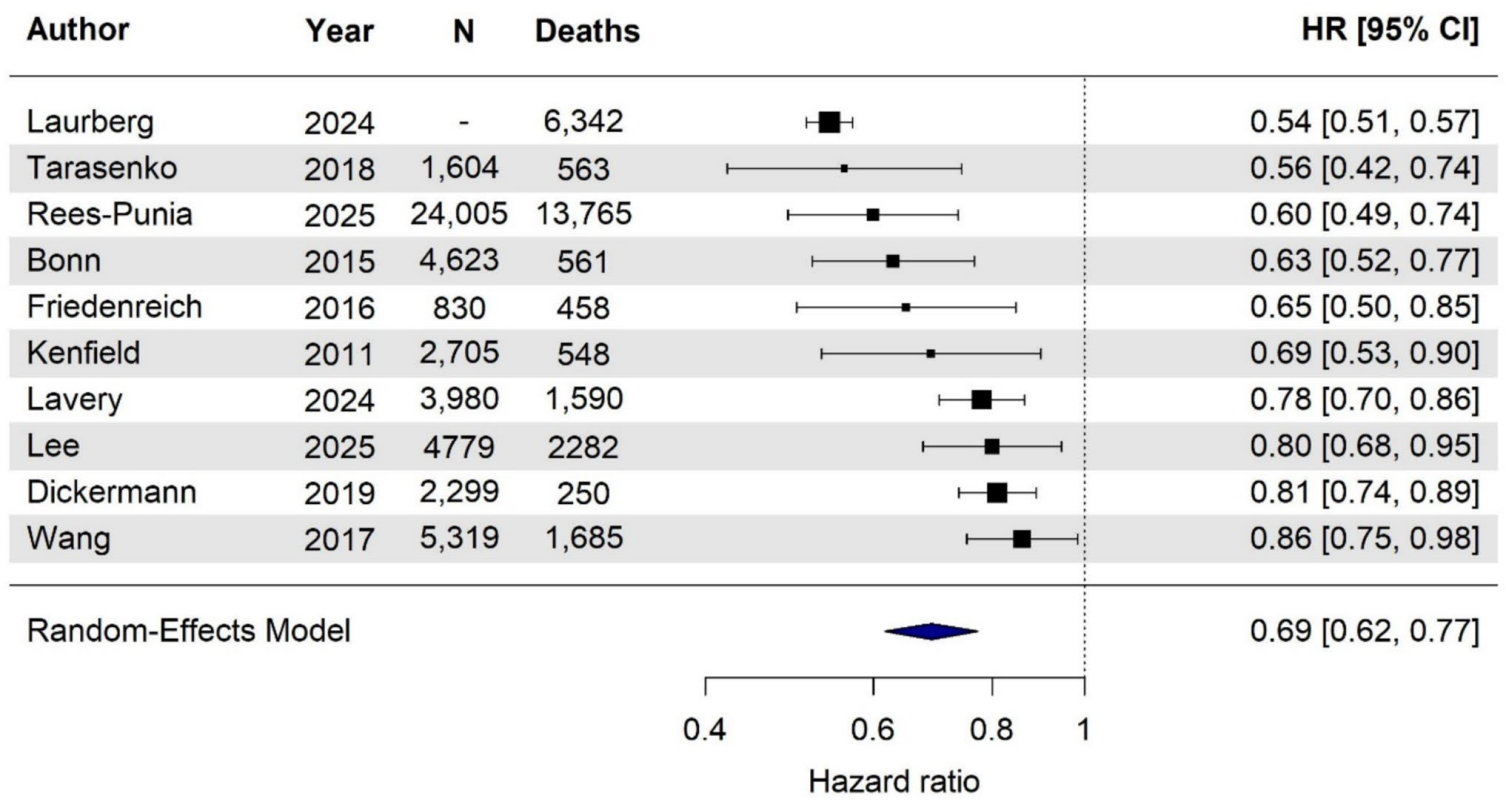
Meta-analysis of higher compared to lower levels of post-diagnosis physical activity and all-cause mortality. *CI* confidence interval, *HR* hazard ratio

Figure 2 shows risk estimates for higher (≥ 7.5 MET-hr/week) compared to lower levels (< 7.5 MET-hr/week) of post-diagnosis physical activity in relation to all-cause mortality.

The pooled analysis of ten studies demonstrated a statistically significant reduction in all-cause mortality. Using a random-effects model, the combined HR was 0.69 (95% CI: 0.62–0.77). Between-study heterogeneity was high (*I*^2^ = 84.7%; *τ*^2^ = 0.025; Q(df = 9) = 100.74; *p* < 0.001). The individual risk estimates ranged from 0.54 (95% CI: 0.51–0.57; [11]) to 0.86 (95% CI: 0.75–0.98; [20]).

To investigate whether study-level characteristics contributed to the heterogeneity observed, a mixed-effects meta-regression was performed including physical activity type, study year and the cutoff for high levels of physical activity as moderators. Residual heterogeneity remained high after adjustment (*τ*^2^ = 0.028; *I*^2^ = 85.9%) and the test for residual heterogeneity was statistically significant (QE(df = 4) = 42.5; *p* < 0.001), indicating that considerable unexplained between-study variability persisted.

The overall moderator test was not statistically significant (QM(df = 5) = 3.69; *p* = 0.595) and the model explained none of the between-study heterogeneity (*R*^2^ = 0%). None of the included moderators meaningfully influenced the association between higher post-diagnosis physical activity and all-cause mortality. Compared to studies assessing MVPA (reference category), studies evaluating moderate physical activity, recreational physical activity, or total physical activity did not differ in their effect sizes (*p* ≥ 0.416). Likewise, neither the year of study (*β* = 0.079–0.148; *p* ≥ 0.709) nor the definition of the physical activity cutoff (*β* =  − 0.224–0.311; *p* ≥ 0.416) was associated with differences in effect size.

An additional meta-analysis restricted to four studies assessing MVPA indicated that higher levels compared to lower levels of post-diagnosis MVPA were associated with a 38% lower all-cause mortality risk. Between-study heterogeneity was high (*I*^2^ = 87.9%; Q(df = 3) = 38.10; *p* < 0.001; τ^2^ = 0.028) *(Supplementary App Figure A1).*

### Prostate cancer-specific mortality

**Fig. 3:**
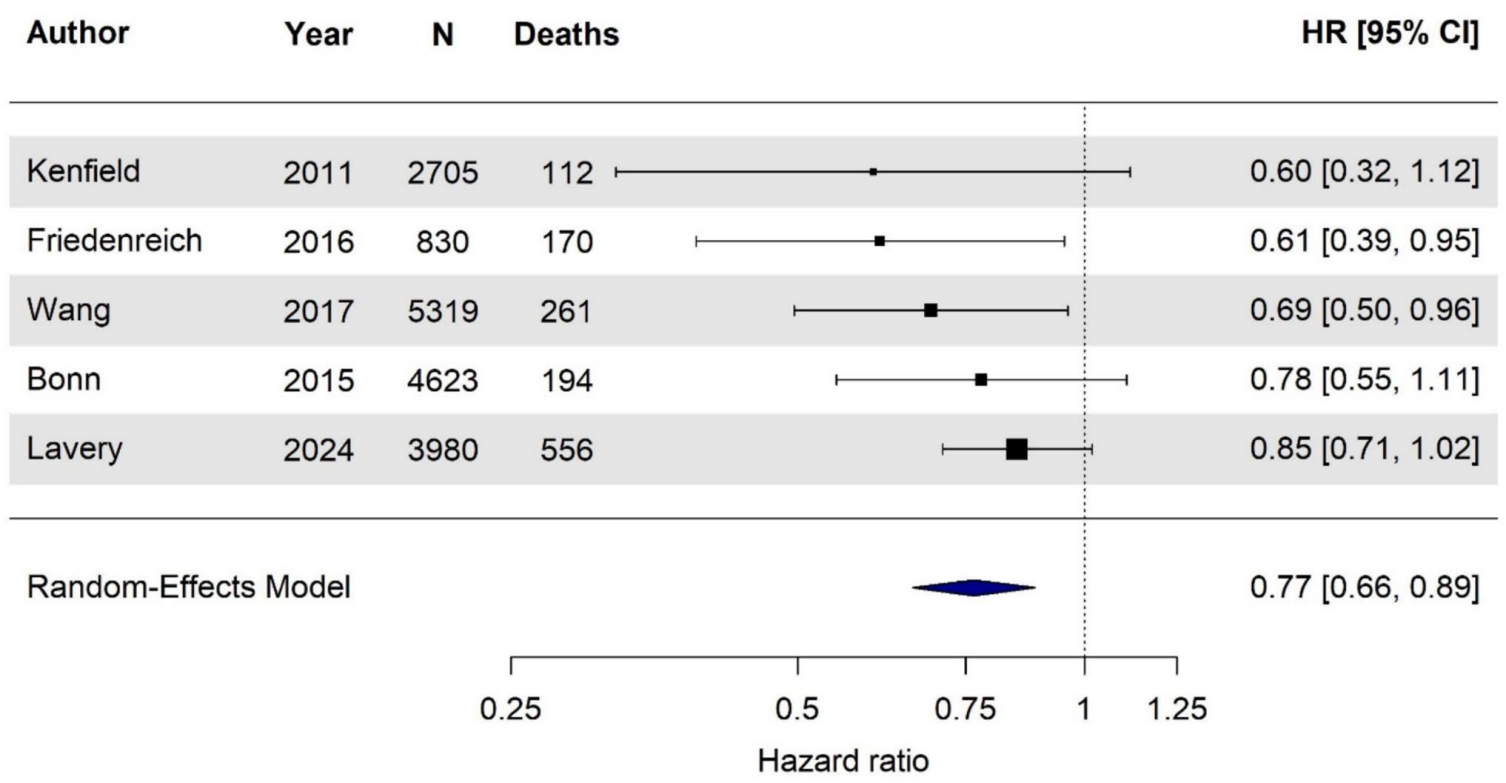
Meta-analysis of higher compared to lower levels of post-diagnosis physical activity and prostate cancer-specific mortality. *CI* confidence interval, *HR* hazard ratio

Five studies reported prostate cancer-specific mortality, reporting 1,293 prostate cancer-related deaths. Meta-analysis of these studies showed a 23% lower mortality associated with higher post-diagnosis physical activity (HR = 0.77; 95% CI: 0.66–0.89) (Fig. 3). Between-study heterogeneity was low (*I*^2^ = 8.7%; *τ*^2^ = 0.0028; Q(4) = 3.23, *p* = 0.52).

### Publication bias

Publication bias was assessed using funnel plots and Egger’s and Begg’s tests. For the primary meta-analysis on all-cause mortality, the funnel plot demonstrated asymmetry *(Supplementary Figure A2)* but the Egger’s regression intercepts showed no statistically significant asymmetry (*p* = 0.541). Begg’s rank-correlation test did not indicate statistically significant small-study effects (*p* = 0.156). Because few studies were included (*n* < 10), publication bias was not assessed for MVPA- and prostate cancer sub-analyses. Funnel plots were generated for descriptive purposes, suggesting asymmetry *(Supplementary Figures A3 and A4).*

## Discussion

We identified ten studies including more than 50,000 men with prostate cancer. Higher levels of post-diagnosis physical activity were associated with lower all-cause and prostate cancer-specific mortality. Our results indicate that men living with prostate cancer benefit from engaging in recommended levels of weekly physical activity, including activities at higher intensities. These findings support physical activity as a complementary strategy for better health after cancer diagnosis.

Our results align with previous smaller meta-analyses showing survival benefits of post-diagnosis physical activity in men with prostate cancer, consistent with our estimates [3, 22]. However, those earlier meta-analyses differ from the present work in several aspects. Rabbani et al. [3] summarized evidence across multiple cancer types and considered a broad lifestyle perspective by additionally examining alcohol moderation, smoking, and dietary changes. Their assessment of post-diagnosis physical activity in men with prostate cancer in relation to all-cause mortality was based on seven studies; analyses of prostate cancer-specific mortality were not performed. Ungvari et al. [22] synthesized evidence across different cancer types, also assessing prostate cancer-specific mortality. However, the authors included studies characterized by substantial heterogeneity in the operationalization of physical activity, as exposure definitions encompassed cardiorespiratory fitness, lifetime physical activity, and post-diagnosis physical activity, thereby limiting the ability to isolate associations between post-diagnosis physical activity and mortality among prostate cancer survivors. In contrast, we applied stricter inclusion criteria to ensure a more homogeneous and clinically interpretable assessment of post-diagnosis physical activity, specifically among men with prostate cancer. By synthesizing data from ten studies, we provide more generalizable evidence on the association between post-diagnosis physical activity and both all-cause and prostate cancer-specific mortality, allowing for a more targeted evaluation of its potential benefits.

Furthermore, we extend these prior findings by assessing the association between post-diagnosis MVPA and mortality. We found that not only higher overall post-diagnosis physical activity but also higher levels of MVPA, which includes vigorous-intensity activity, were associated with markedly lower mortality. Together, these results reinforce the importance of remaining physically active after a diagnosis of prostate cancer.

Several biological pathways may explain the beneficial association of physical activity in improving cancer outcomes. Generally, regular exercise lowers circulating insulin-like growth factors and improves insulin sensitivity. Both are implicated in cancer progression [23]. Physical activity also reduces systemic inflammation through a decrease in C-reactive protein and pro-inflammatory cytokines such as IL-6 and TNF-*α*, particularly following aerobic and combined exercise training [24, 25]. In men with prostate cancer, a randomised preoperative high-intensity trial demonstrated enhanced immune regulation, including increased intra-tumoural NK-cell infiltration [26]. In addition, exercise interventions in prostate cancer survivors undergoing androgen deprivation therapy have consistently been shown to counteract muscle loss and improve metabolic health and overall physical function [27]. These adaptations are closely linked to reductions in systemic inflammation and improved immune competence. Better cardiorespiratory fitness, which is an established predictor of lower cancer mortality [28], may further improve treatment tolerance and overall resilience. Moreover, physical activity modulates sex hormone profiles, and vigorous-intensity exercise in particular has been linked to greater mitochondrial capacity, reduced visceral adiposity and improved endothelial function [29, 30]. These mechanisms are also relevant in prostate cancer aetiology. The consistent inverse association observed across independent cohorts supports the robustness of the relationship between post-diagnosis physical activity and mortality in men with prostate cancer.

An important consideration in prostate cancer survivorship is the heterogeneity of treatment strategies, which may modify the association between post-diagnosis physical activity and mortality. We identified one study that was excluded from the meta-analysis due to the absence of treatment-independent risk estimates, and that reported inverse associations among men receiving surgery or hormone therapy, but not radiation therapy [21]. These differences may reflect variation in treatment-related metabolic and functional impairments, underlying disease severity, patient characteristics influencing treatment selection, or a combination of both. Additional treatment-specific studies are needed to clarify whether associations between post-diagnosis physical activity and mortality differ across prostate cancer treatment regimens.

Our study highlights the applicability of general recommendations from the WHO [6] and the ACSM [31] to the population of men living with prostate cancer. Clinicians should consider integrating structured physical activity counselling, referral to certified exercise oncology specialists, and supervised activity programs into survivorship care. Given the safety profile and broad health benefits of physical activity, promoting activity after cancer diagnosis represents a low-cost, scalable intervention with substantial impact. However, its feasibility may depend on the individual severity of cancer-related symptoms and treatment side effects.

Future research should focus on establishing the optimal type and intensity of exercise interventions for men with prostate cancer. Objective physical activity measurement using wearable devices should complement self-reported data to improve exposure assessment accuracy and reduce recall and social desirability bias. Given the heterogeneity of prostate cancer treatment approaches, future studies should more carefully account for potential confounding by indication, baseline health status, and treatment-related adverse effects when evaluating associations between post-diagnosis physical activity and mortality. Further studies should also examine composite endpoints, such as progression-free survival and recurrence, and evaluate dose–response relationships between physical activity and mortality to define clinically relevant thresholds of benefit and to inform public health guidelines. Expanding research to diverse global populations and tumour subtypes will improve external validity.

### Strengths and limitations

Our study benefits from the prospective cohort designs, ensuring a clearly defined temporal relationship between physical activity exposure and mortality. The study cohorts were generally large and population-based, providing sufficient statistical power and external validity. Reported follow-up durations allowed the evaluation of long-term survival. Physical activity was measured using validated questionnaires and mortality outcomes were derived from national or institutional registries. All studies controlled for confounders such as age, body mass index, smoking, and comorbidities, enhancing internal validity.

However, several limitations need to be considered. All included studies were of observational design, which precludes causal inference despite consistent temporal associations. Physical activity was self-reported, introducing possible recall and social desirability bias. We observed substantial heterogeneity between studies, reflecting possible differences in physical activity assessment methods, timing of measurement, and study populations. Although the magnitude of heterogeneity was high, the direction of association was largely consistent across studies, indicating a robust inverse relationship between post-diagnosis physical activity and mortality. Potential moderators were assessed but did not explain between-study variability. Residual confounding cannot be excluded, particularly regarding diet, psychological factors, or treatment details. Finally, reverse causation remains possible as more active individuals may have had better baseline health.

## Conclusion

Our study shows that both higher volumes and higher intensities of physical activity after a diagnosis of prostate cancer are consistently associated with reduced all-cause and prostate cancer-specific mortality. By synthesising evidence from ten studies, we highlight the important role of physical activity as a modifiable lifestyle behaviour in improving survival. Integrating physical activity recommendations into routine oncological care may represent a low-cost, accessible, and potentially powerful strategy to improve health.

## Supplementary Information

Below is the link to the electronic supplementary material.

Supplementary file1 (DOCX 180 KB)

## Data Availability

No datasets were generated or analysed during the current study.
