# Supplementary material for: Post-diagnosis physical activity in relation to mortality among prostate cancer survivors: a systematic review and meta-analysis
Source: Cancer Causes Control. 2026 Jun 25;37(7):108. doi: 10.1007/s10552-026-02197-2 (PMC13303671; doi:10.1007/s10552-026-02197-2)

**Post-diagnosis Physical Activity in Relation to Mortality among Prostate Cancer Survivors: a Systematic Review and Meta-Analysis**

**Supplementary material**

[Table A1: Study Scores on the New-Castle-Ottawa-Scale](#_Toc224207013)

[Figure A1: Meta-analysis of post-diagnosis moderate-to-vigorous physical activity in relation to all-cause mortality](#_Toc224207014)

[Figure A2: Funnel plot primary analysis on all-cause mortality](#_Toc224207015)

[Figure A3: Funnel plot studies assessing moderate-to-vigorous physical activity](#_Toc224207016)

[Figure A4: Funnel plot studies assessing prostate cancer-sepcific mortality](#_Toc224207017)

**Corresponding author**: Michael J. Stein, Tel.: +49 89 318748819, Mail: michael.stein@helmholtz-munich.de, Department of Epidemiology and Preventive Medicine, University of Regensburg, Regensburg, Germany

### Table A1: Study Scores on the New-Castle-Ottawa-Scale

|  | **Selection** | | | | | **Comparability** | | **Outcome** | | | | **Total** |
| --- | --- | --- | --- | --- | --- | --- | --- | --- | --- | --- | --- | --- |
| **Author, year** | **Representativeness of the exposed cohort** | **Selection of the non-exposed cohort** | **Ascertainment of exposure** | **Demonstration that outcome of interest was not present at start of study** | **x/4** | **Study controls for** | **x/2** | **Assessment of outcome** | **Was follow-up long enough for outcomes to occur** | **Adequacy of follow up of cohorts** | **x/3** | **x/9** |
| Rees-Punia, 2025 | 6 large US cohorts (over 90,000 survivors) | Comparison within the group by activity level | Self-reported via validated questionnaires (various) (listed in their supplementary material) | Assessment earliest 1 year after diagnosis – not possible | 4/4 | Age, race/ethnicity, smoking status, alcohol use, and cancer treatment and stage | 2/2 | National Death Index | 10,9 years | Nearly complete | 3/3 | 9/9 |
| Lavery, 2024 | 11,000 US cancer survivors (4,000 PCS) | Comparison within the cohort by activity level | Standardized questionnaire (SQX) | Study entry 6 months after SQX administration (participants who died before this time point were excluded) | 4/4 | Time between diagnosis and SQX, age at SQX, sex, race/ethnicity, PLCO randomization group, cancer stage at diagnosis, primary cancer treatment ( prostatectomy, radiation and hormone treatment without prostatectomy, radiation without prostatectomy or hormone treatment, hormone treatment without prostatectomy or radiation, other ablative treatment, and no known treatment with curative intent), pack-years of cigarette smoking, body mass index (BMI) at study entry, PLCO randomization year, and independent comorbidity history of arthritis, chronic bronchitis, diabetes, diverticulitis/diverticulosis, emphysema, gallbladder stones or inflammation, colon-related comorbidity, coronary heart disease/history of heart attack, hypertension, liver-related comorbidities, osteoporosis, and stroke | 2/2 | National Death Index (NDI) and PLCO adjudication | 11,6 years | No losses reported | 3/3 | 9/9 |
| Tarasenko, 2018 | NHIS cancer survivors, minimum 3 years | Comparison within the cohort by activity level | Self-reported via NHIS/NCHS questionnaire | Deaths could not occur before study entry | 4/4 | Age, sex, race/ethnicity, education level, marital status, and insurance status, self-rated health, activity limitations, smoking status, BMI categories, number of comorbid conditions, and age at first cancer diagnosis | 2/2 | NHIS and National Death Index (NDI) data | 16,8 years | 100% | 3/3 | 9/9 |
| Kenfield, 2011 | HPFS (Health Professionals Follow-up Study) US male health professionals | Comparison within the cohort by activity level | Self-reported via weekly questionnaires in MET-hr/week (questionnaire based on validated instrument) | Deaths could not occur before study entry | 3/4 | Age, months since diagnosis, clinical stage, Gleason score, treatment, parental history of myocardial infarction at age 60 years or younger, high blood pressure, elevated cholesterol, diabetes status, smoking status, body mass index, alcohol intake, comorbidities (myocardial infarction, coronary artery bypass, coronary angioplasty, stroke, Parkinson's disease, and emphysema, chronic bronchitis), prediagnosis physical activity | 2/2 | Adjudicated by study physicians (medical records and death certificates) | 9,7 years | 98% | 3/3 | 8/9 |
| Friedenreich, 2016 | Alberta Cancer Registry (ACR) data | Comparison within the cohort by activity level | Comparison within the cohort by activity level | Prospective mortality assessment | 4/4 | Age at diagnosis, overall stage (II, III, III/IV, IV), treatment (prostatectomy, radiation therapy, hormone therapy), Gleason score, PSA level, region (urban vs rural), number of times had PSA test done (never/once/twice or more), total pack-years of smoking at diagnosis, postdiagnosis total pack-years of smoking, prediagnosis total physical activity, postdiagnosis comorbidity (Charlson Comorbidity Score), time to any first recurrence/progression of prostate cancer | 2/2 | Alberta Health registration file | 15,5 years | 100% | 3/3 | 9/9 |
| Wang, 2017 | HPFS | Comparison within the cohort by activity level / sedentary time | Self-reported via questionnaire (validation missing) | Prospective mortality assessment | 3/4 | Age at diagnosis, race, calendar year of diagnosis, tumor extent, nodal involvement, Gleason score, initial treatment, history of pre-diagnosis prostate-specific antigen testing, post-diagnosis diabetes history, post-diagnosis cardiovascular disease history, post-diagnosis other cancer history, post-diagnosis body mass index, pre-diagnosis alcohol consumption, post-diagnosis smoking status, pre-diagnosis intake of red meat and processed meat, fish, and fruit and vegetables, post-diagnosis leisure time sitting | 2/2 | National Death Index | 8,4 years | Nearly complete | 3/3 | 8/9 |
| Dickermann, 2019 | HPFS | Comparison within the cohort by activity level | Self-reported via validated questionnaire* (see Chasan-Taber et al., 1996) | Deaths measured only after exposure assessment | 4/4 | Age, parental history of myocardial infarction before age 60 years, primary treatment, clinical stage, Gleason grade, and prostatespecific antigen level at diagnosis, prebaseline values of BMI, smoking history, and vigorous and moderate physical activity; for the following time-varying covariates: vigorous and moderate physical activity, BMI, the development of conditions excluded at baseline (functional impairment, metastasis, myocardial infarction, stroke, congestive heart failure, amyotrophic lateral sclerosis) | 2/2 | Personal research / National Death Index | 10 years | Minimal losses 93,4% complete | 3/3 | 9/9 |
| Bonn, 2015 | NCR Sweden (National Cancer Registry of Sweden) | Comparison within the cohort by activity level | Self-reported via validated questionnaire (see Norman et al., 2001) | Deaths measured only after exposure assessment / occurrence | 4/4 | age at diagnosis (5-year intervals), Gleason score (<6, 6, >6), primary treatment, serum PSA (continuous), BMI at diagnosis (<25, 25–<30, 30 kg/m2), and weight change (>5% increase, >5% decrease, no change) | 2/2 | Swedish Cause-of-Death Registry | 4,7 years | No relevant losses | 3/3 | 9/9 |
| Laurberg, 2024 | NDR Sweden  (National Diabetes Register of Sweden) – selective but large | Comparison within the cohort by activity level | Extracted from NDR registry | Deaths measured only after exposure assessment / occurrence | 3/4 | Sex, age, Country birth, educational level, diabetes duration and civil status, smoking, physical activity level, BMI, systolic blood pressure, HbA1c, albuminuria, LDL-cholesterol | 2/2 | Causes-of-death-register | 5,8 years | No relevant losses | 3/3 | 8/9 |
| Lee, 2025 | HPFS | Comparison within the cohort by activity level | Self-reported via validated questionnaire (every 2 years) | Prospective mortality assessment, outcome absent at baseline | 4/4 | Age at diagnosis, stage (T1, T2, T3a), Gleason score (<7, 7,≥8, missing), treatment (radical prostatectomy, radiation, hormones, other), prostate-specific antigen level at diagnosis (<4, 4-9, 10-19.9,≥20 ng/mL, missing), smoking (never, former/quit>10 years ago, former/quit≤10 years ago, current), diabetes mellitus (yes or no), elevated cholesterol (yes or no), high blood pressure (yes or no), parental history of myocardial infarction before age 60 years, and comorbid condition (yes or no; conditions included myocardial infarction, coronary artery bypass or angioplasty, stroke, emphysema or chronic obstructive pulmonary disorder, and Parkinson disease) | 2/2 | National Death Index, reports from next of kin, medical records / death certificates | 15 years | 98% | 3/3 | 9/9 |

### Figure A1: Meta-analysis of post-diagnosis moderate-to-vigorous physical activity in relation to all-cause mortality


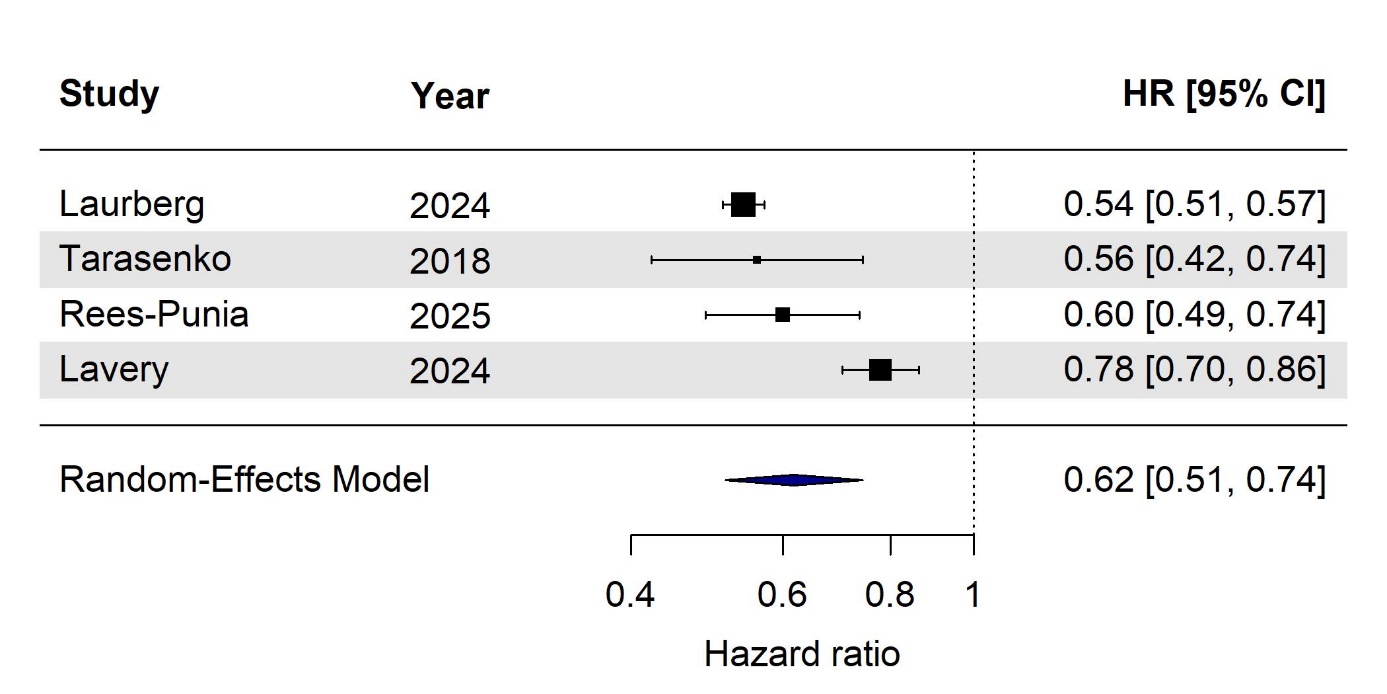


### Figure A2: Funnel plot primary analysis on all-cause mortality


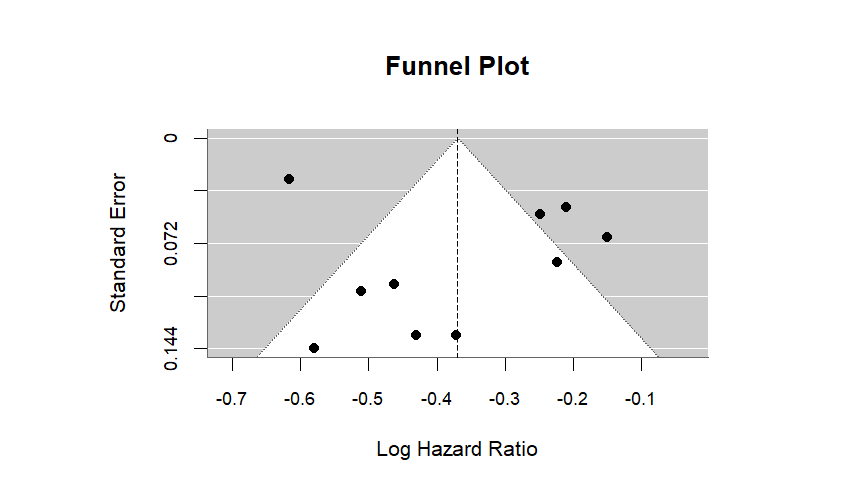


###
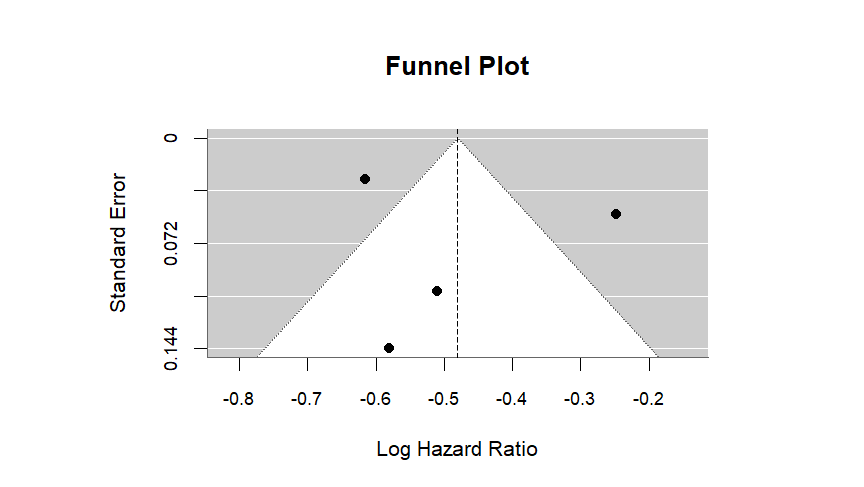
Figure A3: Funnel plot studies assessing moderate-to-vigorous physical activity

### Figure A4: Funnel plot studies assessing prostate cancer-sepcific mortality


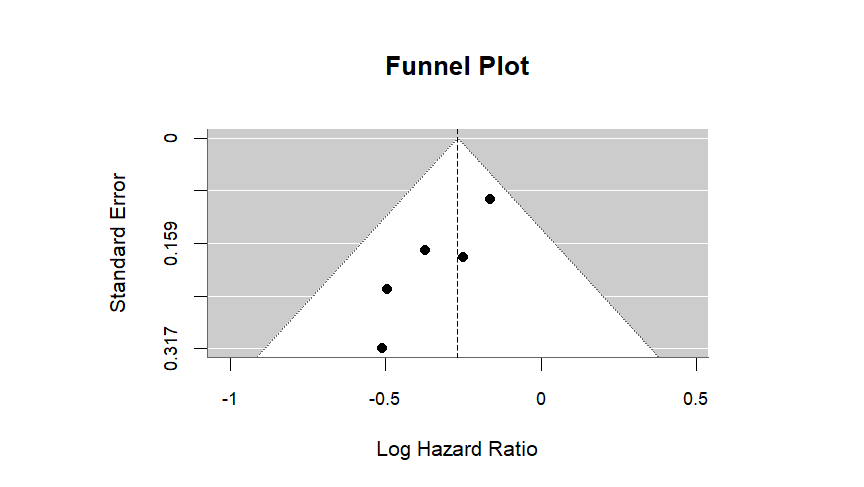

Supplement: Supplementary file 1 — Supplementary file1 (DOCX 180 KB) [file 10552_2026_2197_MOESM1_ESM.docx]
